# Flat versus Simulated Mountain Trail Running: A Multidisciplinary Comparison in Well-Trained Runners

**DOI:** 10.3390/ijerph20065189

**Published:** 2023-03-15

**Authors:** Kristina Skroce, Simone Bettega, Samuel D’Emanuele, Gennaro Boccia, Federico Schena, Cantor Tarperi

**Affiliations:** 1 Faculty of Medicine, University of Rijeka, 51000 Rijeka, Croatia; 2 Department of Neuroscience, Biomedicine and Movement Sciences, University of Verona, 37131 Verona, Italy; 3 Department of Clinical and Biological Sciences, University of Turin, 10124 Turin, Italy

**Keywords:** energy expenditure, running, endurance training, electromyography

## Abstract

This paper compares cardiopulmonary and neuromuscular parameters across three running aerobic speeds in two conditions that differed from a treadmill’s movement: flat condition (FC) and unpredictable roll variations similar to mountain trail running (URV). Twenty well-trained male runners (age 33 ± 8 years, body mass 70.3 ± 6.4 kg, height 1.77 ± 0.06 m, $\dot{V}$O_2_max 63.8 ± 7.2 mL·kg^−1^·min^−1^) voluntarily participated in the study. Laboratory sessions consisted of a cardiopulmonary incremental ramp test (IRT) and two experimental protocols. Cardiopulmonary parameters, plasma lactate (BLa^−^), cadence, ground contact time (GT) and RPE values were assessed. We also recorded surface electromyographic (sEMG) signals from eight lower limb muscles, and we calculated, from the sEMG envelope, the amplitude and width of peak muscle activation for each step. Cardiopulmonary parameters were not significantly different between conditions ($\dot{V}$O_2_: *p* = 0.104; BLa^−^: *p* = 0.214; HR: *p* = 0.788). The amplitude (*p* = 0.271) and width (*p* = 0.057) of sEMG activation peaks did not change between conditions. The variability of sEMG was significantly affected by conditions; indeed, the coefficient of variation in peak amplitude (*p* = 0.003) and peak width (*p* < 0.001) was higher in URV than in FC. Since the specific physical demands of running can differ between surfaces, coaches should resort to the use of non-traditional surfaces, emphasizing specific surface-related motor tasks that are normally observed in natural running environments. Seeing that the variability of muscle activations was affected, further studies are required to better understand the physiological effects induced by systematic surface-specific training and to define how variable-surface activities help injury prevention.

## 1. Introduction

It is well known that running has a significant impact on health and overall well-being, and it is feasible to include it in everyday life [1]. In the last couple of years, breaking the barriers of human performance in road running has been the central focus of many researchers [2,3]. At the same time, off-road running events have become increasingly popular, and participation has grown substantially with many new races appearing around the world [4,5].

The exponential growth of participation in off-road races is likely due to the greater appeal of these competitions compared to road and track events [6]. Environmental characteristics and less impactful running surfaces enhance runners’ perception of the running environment more than intrapersonal factors such as runners’ motives and attitudes [7,8]. 

However, road and off-road running performance models are different in both physiological and biomechanical demands. Off-road running exposes the body to unfamiliar and often irregular stresses. Previous studies documented that running on natural surfaces requires greater metabolic energy expenditure than running on smooth hard surfaces [9,10,11,12]. The level of energy expended in these terrain conditions is likely to fluctuate, even when the running speed is constant. Moreover, it was shown that off-road running elicits a heart rate response that varies with the changing demands of the surface, vegetation and gradient [13]. On the other hand, the running surface can affect running biomechanics [14], as runners adapt their lower limb kinematics to reduce the variability of impact forces depending on the surface [15]. Additionally, trail running significantly elevates the feet’s mediolateral acceleration compared to treadmill running due to the variability of the landing surface [16]. Running on a trail significantly modifies running patterns, and it is likely that the trajectory should be adjusted with more lateral deviations to find the easiest (or safest) path when obstacles are located on the trail’s surface [16]. 

Running on trails requires the rapid cognitive processing of the environment, responsiveness to variations in the ground surface, the avoidance of obstacles and adjustment to elevations in terrain. To maintain stability, runners use anticipatory, predictive and reactive balance control strategies [17,18]. 

The motor control of running requires coordination between the skeletal and neuromuscular systems. For this purpose, surface electromyography (sEMG) represents the neuromuscular response to the different requirements in human locomotion, such as different speeds and both predictable and unpredictable surfaces [19,20]. The metabolic cost associated with various types of muscle contractions remains a valid explanation for the high and low costs of uphill and downhill running, respectively, from −20 to +45% grades [21,22]. 

However, off-road running takes place in more complex environments and conditions, which imply a high number of complex unpredictable variables. This can make off-road running a high-risk discipline for lower limb extremity injuries [23], with the majority of acute and chronic musculoskeletal injuries occurring in the knee and ankle [18]. Even though the surface itself does not represent the primary cause of injury, deficits in the neuromotor control of the lower extremity joints and the abnormal motion of loaded joints are potential mechanisms. 

Being able to effectively move over diverse terrain conditions has been an integral part of this performance model, and it is fundamental for injury prevention [18].

The application of a consistent set of neural control elements during perturbed steady-state locomotion was defined, but with modifications of the basic activation patterns. This indicated a transition from an accurate to a more robust movement control in the presence of continuously variable perturbations [20]. 

However, little is known regarding the effects that different running surfaces impose on the variability of lower limb muscle activity and how they impact cardiorespiratory parameters. Minetti et al. [24] identified methodological issues that hampered the accurate reproduction of outdoor conditions in the laboratory. Lately, a modified treadmill has been used to produce such surface conditions in a laboratory environment [25].

**Figure 1:**
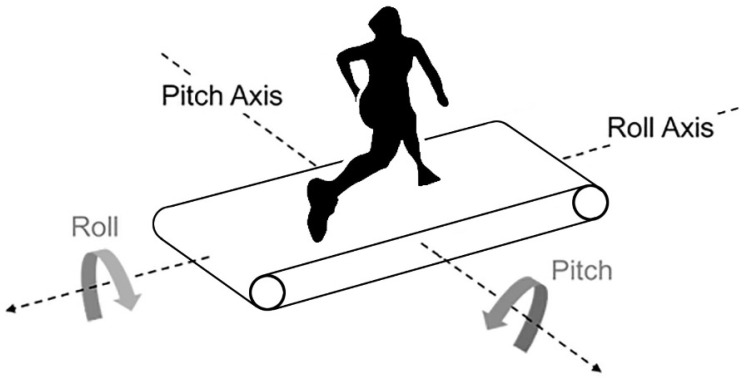
Inclinations allowed by the treadmill used in the study for URV and FC conditions.

Therefore, the aims of this study were to compare the cardiopulmonary and neuromuscular parameters of well-trained male athletes running across three speeds (8, 10 and 12 km·h^−1^) and in two conditions that differed from a treadmill’s movement: flat condition (FC) and with unpredictable roll variations (URV, see Figure 1). We hypothesized that the URV would induce greater neuromuscular activation with, consequently, greater metabolic and cardiopulmonary responses compared to FC. 

## 2. Methods

### 2.1. Participants

Twenty well-trained male runners voluntarily participated in the study. The main anthropometric and physiological characteristics are reported in Table 1. Before taking part in the study, participants provided informed written consent. The Institutional Review Board of the University of Verona approved the study’s protocol (n.13.R1/2021), and it was conducted according to the ethical standards of the Helsinki Declaration.

### 2.2. Experimental Design

The study comprised three visits separated by 48 h for each subject. Visit 1 consisted of a cardiopulmonary incremental ramp running test (IRT) until volitional exhaustion. Visit 2 and 3 consisted of experimental protocols that were different from the treadmill’s movement pattern. The participant’s metabolic energy consumption rates, blood lactate (BLa^−^), sEMG of eight muscles in the right lower limb, cadence, ground contact time (GT) and values from the ratings of the perceived exertion scale (RPE) at three different aerobic speeds were assessed in two conditions: FC and URV. During the FC, the treadmill was in a standard, neutral position and inclined at 1% during IRT and URV. During URV, the lateral inclination of the treadmill varied unexpectedly. Carbon-plated shoes were not used in any of the experimental or testing sessions.

One month prior to incremental testing, participants were familiarized with the RPE (Borg centiMax^®^ Scale, 0–100) [26] and the setting of the URV condition. 

### 2.3. Testing Procedures

During the first visit, anthropometric data were collected, and participants carried out an IRT starting from 8 km·h^−1^ and speeding up to 0.5 km·h^−1^ every minute until volitional exhaustion. All tests were carried out on a motorized treadmill (ReaxRun, Reaxing, Milano, Italy), and the inclination was kept fixed at 1% throughout the test [27].

During the second and the third visit, participants completed a 6-minute trial for each speed at 8, 10 and 12 km·h^−1^ in both FC and URV experimental protocols. The speed and condition of the protocols were assigned in random order once the IRT was completed. For the URV condition, a computer-generated randomized roll oscillation routine was made according to the following criteria: (I) Treadmill lateral inclination ranged at +7°/−7° relative to the horizontal, and (II) each position was maintained between 1 and 3 s. The same routine was repeated for all running speeds and subjects. Subjects were strongly encouraged to replicate their diet, sleep and training pattern for all laboratory visits. 

### 2.4. Cardiopulmonary Measurements

During all visits, oxygen consumption ($\dot{V}$O_2_), carbon dioxide production ($\dot{V}$CO_2_), minute ventilation ($\dot{V}$E), respiratory exchange ratio (RER) and heart rate (HR) were recorded with a breath-by-breath analyzer (K5, Cosmed, Roma, Italy) that was previously set and calibrated according to the manufacturer’s instructions. 

### 2.5. Gait Parameters

GT and cadence were collected by an inertial measurement unit placed on the right shoe (Stryd Summit Powermeter, firmware 1.2, Boulder, CO, USA) throughout the 6 min running bouts. 

### 2.6. Surface Electromyography

During visits 2 and 3, 8 pairs of bipolar electrodes (24 × 24 mm; CDE-C, OTBioelettronica, Torino, Italy) were placed, according to the SENIAM guidelines [28], on the following muscles of the right lower limb: bicep femoris (BF), gastrocnemius medialis (GM), semitendinosus (ST), peroneus longus (PL), soleus (SOL), tibialis anterior (TA), vastus lateralis (VL) and vastus medialis (VM). The distance between electrodes was 20 mm. Before the placement of electrodes, the skin was prepared, hair was shaved, and an abrasive paste (Nuprep, Weaver and company, Aurora, CO, USA) was used to reduce the impedance. In the end, the skin was cleaned with alcohol [29]. In order to avoid movement-induced artifacts, the wires connected to the electrodes were well secured with adhesive tape (Hypafix, 5 × 10 cm; Leukoplast, Hull, UK). sEMG signals were recorded (OTBiolab software v. 1.5.5, OTBioelettronica, Torino, Italy) during the entire running protocol at a sampling frequency of 2048 Hz (gain 200 *V*/*V*, bandwidth 10 ÷ 500 Hz, A/D converter resolution 16 bit) using four wireless probes (DueLite, OTBioelettronica, Torino, Italy).

### 2.7. Data Analysis

#### 2.7.1. Cardiopulmonary Analysis

Following visit 1, both the gas exchange threshold (GET) and the respiratory compensation point (RCP) were independently determined by three blinded experimenters. The average of the three values was used for analysis as long as all estimates were within 200 mL·min^−1^. If one of the experimenter estimates was not within 200 mL·min^−1^, an average of the two in closest agreement was used. GET was determined by visual inspection as the $\dot{V}$O_2_ at which CO_2_ output ($\dot{V}$CO_2_) began to increase out of proportion in relation to $\dot{V}$O_2_, with a systematic increase in the minute ventilation $\dot{V}$E-to-$\dot{V}$O_2_ relationship and end-tidal PO_2_, whereas the ventilatory equivalent of $\dot{V}$CO_2_ ($\dot{V}$E/$\dot{V}$CO_2_) and end-tidal PCO_2_ were stable [30]. RCP was determined as the point where end-tidal PCO_2_ began to fall after a period of isocapnic buffering. This point was confirmed by examining $\dot{V}$E/$\dot{V}$CO_2_ plotted against $\dot{V}$O_2_ and by identifying the second breakpoint in the $\dot{V}$E-to-$\dot{V}$O_2_ relationship. $\dot{V}$O_2peak_ was defined as the highest 20 s $\dot{V}$O_2_ computed from a rolling average, and S_max_ was defined as the speed achieved at the termination of the IRT. Breath-by-breath $\dot{V}$O_2_ data were edited on an individual basis: Aberrant data that lay 3 standard deviations (SD) from the local mean were removed, and trials were linearly interpolated on a second-by-second basis, time-aligned such that time “zero” represented the onset of exercise (i.e., the onset of constant-load or IRT protocol), and the trials were averaged into 5 s and 30 s time bins. HR was collected using radiotelemetry (SP0180 Polar Transmitter, Polar Electro Inc., Kempele, Finland) and calculated over the duration of each breath.

During visits 2 and 3, $\dot{V}$O_2_ and $\dot{V}$CO_2_ were measured, and the rate of metabolic energy consumption was calculated over the last 3 min of each trial, using the dI Prampero equation [31]. Lactate samples (Biosen C-line, EKF Diagnostics, Barleben, Germany) from the ear lobe were collected after every 6-minute running bout.

#### 2.7.2. Surface Electromyography

sEMG signal analyses were carried out with a custom-written script in MATLAB (v. R2020b; Mathworks, CO, USA). Raw sEMG signals were first filtered with a Butterworth 30–400 Hz. We then identified 300 cycles of uncorrupted signals for each subject, speed (8, 10 and 12 km·h^−1^) and condition (FC and URV). After that, we calculated the sEMG envelopes with rectification and low pass filtering (cut-off 10 Hz, 4th order Butterworth). To characterize the muscle activation of each step, we adopted the *findpeaks* MATLAB function to identify the local maxima (peaks) in the envelopes of sEMG signals, their amplitude (peaks_amplitude (µV)) and their width calculated at mid-amplitude (peaks_width (ms)). The average and coefficient of variation (CoV_peaks_height (%), CoV_peaks_width (%) and CoV_peaks_intervals (%)) in each parameter were also calculated over the 300 cycles.

### 2.8. Statistics

Based on Voloshina’s metabolic data [25], the minimal sample size (n° = 19) was calculated a priori. We also verified the sample size a posteriori based on the mean CoV_Width as the representative result. In both cases, the alpha error was set to 0.05, and beta was set to 0.8, obtaining a minimum number of subjects equal to 16. G*Power (Version 3.1.9.5) software was used to estimate the sample size. The effect size (ES) was calculated as Cohen’s d for paired *t*-test comparisons (small = 0.2; medium = 0.5; large = 0.8) and partial eta squared (η_p_^2^) for repeated measures ANOVA (small = 0.01; medium = 0.06; large = 0.14). We analyzed the data using GraphPad Prism 8 (v.8.2.0) with a significance set at *p* ≤ 0.05. The distribution normality of all parameters was tested via the Shapiro–Wilk test. In case data collected were normally distributed, repeated measures ANOVA was used to assess the impact of the condition. Otherwise, the Friedman test was used (Bla^−^ and RPE). For the sEMG signals, when the distribution was not normal, the data were log-transformed before performing Student’s *t*-test or ANOVA analysis. For cardiopulmonary and gait parameters, two-way repeated measures ANOVA was used to assess the impact of the condition (FC vs. URV) and the speed (8, 10 and 12 km·h^−1^). For the sEMG signals, a three-way repeated measures ANOVA was performed between muscles, speed and condition. Post hoc tests were corrected with the Bonferroni correction.

## 3. Results

### 3.1. Metabolic and Cardiopulmonary Parameters

Mean data (±standard deviation (SD)) for $\dot{V}$O_2_, BLa^−^, HR, cost of running (Cr), RPE, cadence and GT are reported in Table 2. In general, the increasing levels of metabolic demands across different speeds were accompanied by higher cardiopulmonary output and lactate levels. Indeed, as the speed increased, $\dot{V}$O_2_ (F(2,32) = 2.435, *p* = 0.104), BLa^−^ (F(2,34) = 1.616, *p* = 0.214) and HR (F(2,34) = 0.239, *p* = 0.788) increased. However, the metabolic responses were not significantly different between conditions ($\dot{V}$O_2_: *p = 0.104;* BLa^−^: *p = 0.214*; HR: *p* = *0.788*). Cr remained constant across speeds and conditions (F(2,34) = 27.92, *p* = 0.075).

### 3.2. Gait Parameters

With the speed increase, cadence (F(2,32) = 0.665, *p* = 0.521) also increased as well while GT decreased (F(2,32) = 0.138, *p* = 0.872). However, none of these variables showed differences between conditions. Moreover, the variability of cadence (F(2,32) = 0.062, *p* = 0.940) and GT (F(2,32) = 2.688, *p* = 0.084) did not show any significant difference and were not affected by conditions.

### 3.3. EMG Data

**Figure 2:**
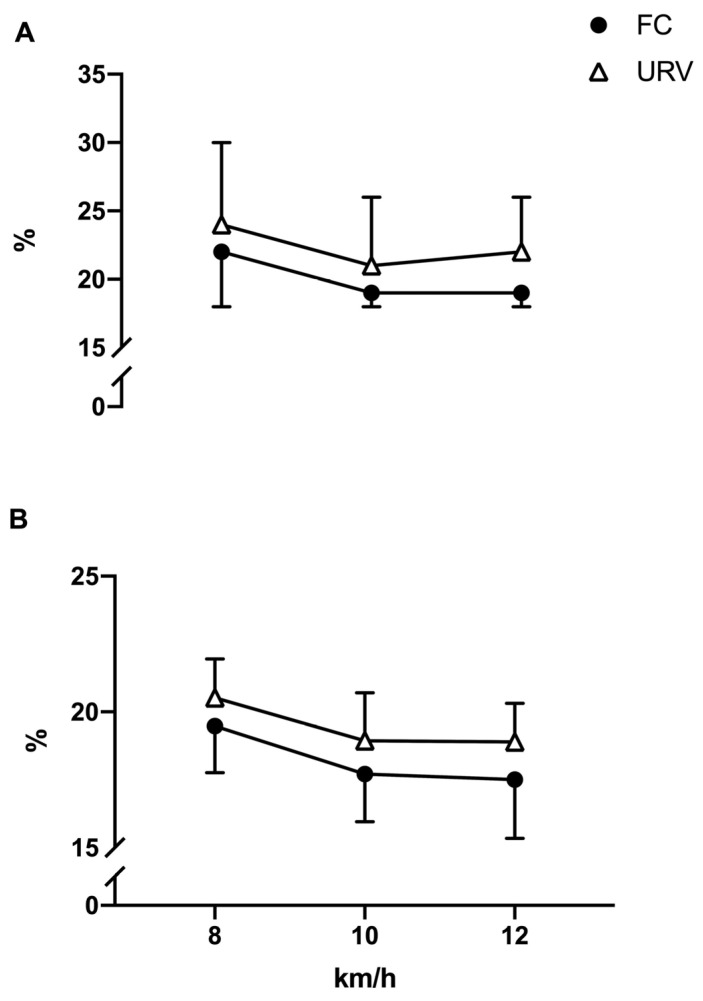
The coefficients of variation of peaks amplitude (**A**) and peaks width (**B**) for FC and URV conditions.

As the speed increased, peaks_amplitude also increased (F(2,38) = 9.765, *p* = 0.003, η_p_^2^ = 0.339) while peaks_width decreased (F(2,38) = 1.746, *p* < 0.001, η_p_^2^ = 0.641). However, the sEMG response was, on average, not different between conditions, and each muscle group responded similarly in the two conditions. Indeed, three-way ANOVA did not show any statistically significant interaction between conditions and muscles for peaks_amplitude (F(7133) = 1.269, *p* = 0.271) and peaks_width (F(7133) = 2.019, *p* = 0.057) (see Figure 2).

Conversely, the variability of sEMG estimates was significantly affected by the conditions, as CoV_peaks_amplitude (F(1,19) = 11.448, *p* = 0.003, η_p_^2^ = 0.376) and CoV_peaks_width (F(1,19) = 19.188, *p* < 0.001, η_p_^2^ = 0.502) were higher in URV than in FC. As the muscle groups never emerged in a statistically significant interaction with conditions or speed, for concision and clarity in Figure 2, we report the results with all muscle groups merged (see Table S1: results for each muscle and condition).

Moreover, the muscle-by-muscle analysis showed a significant difference between two conditions only in BF peaks_width at 10 km·h^−1^ (F(2, 38) = 9.359, *p* = 0.002, η_p_^2^ = 0.330) and in GM peaks_amplitude at 12 km·h^−1^ (F(2,38) = 4.977, *p* = 0.034, η_p_^2^ = 0.208).

### 3.4. Psychological Parameters

Significant differences were not observed between experimental conditions (FC and URV) for the RPE values (see Table 2).

## 4. Discussion

The transversally inclined surface treadmill can represent a novel way of training that simulates variable terrain when outdoor-specific training is not a feasible option. Previous studies demonstrated how cardiopulmonary responses vary on “irregular surfaces” in the form of modified treadmills and natural surfaces [12,25,32,33]. Changes in mechanical work performed by muscles during walking and running on uneven terrain may explain nearly half of the increase in metabolic cost from the transition from smooth to uneven surfaces [25,33]. Moreover, one’s individual capacity to quickly adapt to mechanical challenges relating to trail running is very important in order to put in place the right counteractive strategies needed to promote safe motion. Studies on side-sloped paths are still not represented enough in the scientific literature.

In this study, we performed a comprehensive assessment of running under unpredictable roll variations, using novel equipment, in order to detect any possible increase in physiological demands compared to classic flat running. For this purpose, cardiopulmonary, neuromuscular and psychological responses were measured across three speeds in two running conditions: FC and URV. In our data, in accordance with the results found by Voloshina et al. [25], the step-by-step variabilities (i.e., the coefficient of variation) of sEMG peaks and width were greater under URV. The movement of the foot landing on the treadmill with variable heights (step-in-flight and slope in our study; steps in the others) obviously requires adjustments between the steps, which produces increased electromyographic variabilities. This is well discussed by Martino et al. [34], who suggest that the nervous system adopts the strategy of prolonging the duration of basic muscle activity patterns to cope with unstable conditions. This may be explained by cerebellum activity, which seems to be designated to play a role in balance and foot loading control [35,36]. Moreover, sensory information related to the gait is integrated by the cerebellum to finetune locomotor responses and muscle activation patterns. Unlike previous studies, we did not find greater sEMG activation in the muscles investigated except in GM peaks_amplitude at 12 km·h^−1^ and in BF peaks_width at 10 km·h^−1^ (see supplementary material). We believe that this is because the locomotion in our sideways tiltable treadmill is completely free, and consequently, the subject is able to control their posture in a natural way. We trigonometrically calculated the surface height variation in the central positions of the treadmill (where the foot supports occur), obtaining a value of ±2.8 cm with respect to the height corresponding to the horizontal position. This is similar to the limit values used in Voloshina’s studies [25,33]. Nevertheless, the “contact altitude” in our treadmill should be considered as a continuous variable, unlike the discrete one (such as the steps that characterize the treadmill) used previously. In other words, the runners in our study model must make adjustments corresponding to the maximum value of the altitude variation only in some extreme cases, while in all other cases, these adjustments are minor and sometimes imperceptible. This, as a potential reduction in the stimulus, could be considered a limit; however, it could also be considered a novel method for potentially reducing injury risks and increasing the variability of the stimuli. The latter was in fact demonstrated in an increased sEMG variability. Off-road running has unique characteristics due to the environmental condition in which races are held, which also makes this type of running a high-injury-risk activity. Heater et al. suggested that safe trail running participation can also be achieved on similar terrain [18]. We think that URV running could be a useful and safe tool for stimulating lower limb muscle variability, which leads to useful adaptations for outdoor running. Moreover, fatigue creates a myriad of biomechanical problems [18]. The redistribution of muscle activity or the change in the profile of muscle activity can occur with fatigue [37,38]. Running through fatigue can exacerbate the probability of getting injured. Therefore, given the changes observed in muscle activity, exercising on different surfaces may promote the improvement of postural control needed to adapt to rapidly changing environments and sustain injury prevention at the onset of fatigue.

We expected that postural control, which is necessary to manage the variable inclination of the surface, would induce greater sEMG activation and produce an increase in the energy expenditure of locomotion due to increased cardiopulmonary activity as well as perceived exertion. In our data, cardiopulmonary and metabolic demands did not increase in the URV compared to the FC. Indeed, $\dot{V}$O_2_, HR and Cr were similar under flat and rolling conditions. This is in line with the neuromuscular responses measured with sEMG data: The activation profiles of each muscle did not increase, on average, under rolling conditions. Indeed, both the amplitude and the width of activation peaks of the most relevant muscles of the lower limbs were similar in both conditions. The cadence and GT during unpredictable lateral inclination did not show any average modifications in the experimental condition. This might be the cause of the contrasting results that we found compared to the previous studies where the change in the contact surface type itself led to increased neuromuscular activation. It should also be considered that in our experimental setup, unlike in other studies, the inclination of the support surface can change even during the foot contact itself. This factor can also contribute to sEMG variability. Finally, it is useful to consider that while we analyzed running at 8, 10 and 12 km·h^−1^, Voloshina et al. [25] analyzed the speed of locomotion around 8.2 km·h^−1^, and the ground times are therefore different. This can also produce differences in electromyographic and metabolic measurements. On the other hand, Gantz and Derrick [39] analyzed a speed spectrum that was more similar to ours, obtaining an increase in the bioenergetic cost of locomotion. However, the authors declare that the increased values in biomechanics parameters was due to the uneven running terrain. They assume that runners adopted different techniques in order to prevent injuries, since at contact with the ground, the foot can be impacted by block height variation.

A substantial amount of evidence describes an increase in Cr while walking or running over irregular terrain in the form of modified treadmills and natural surfaces [9,25,32,33,40]. Gantz and Derrick [39] used a wooden block attached to the treadmill and found an increase of 10% in the rate of oxygen consumption on the irregular surface compared to the smooth surface. Changes in mechanical work performed by muscles during walking and running on uneven terrain may explain nearly half of the increase in metabolic cost from smooth to uneven surfaces [25,33]. The fact that the present study did not detect any increase in metabolic or neuromuscular demands can be ascribed to the fact that the perturbations imposed on running kinematics were “too smooth” to induce a noticeable augmented physical demand. The treadmill oscillated laterally by 10.4° s^−1^, and the surface was smooth. This could have allowed the runners to adopt very similar running mechanics to those usually performed without any noticeable disturbance. The present results could be explained even better by the fact that our participants were well-trained runners. They are used to adapting their running technique to irregular terrains and can do so more effectively than non-trained participants. Slater described how less experienced runners have different biomechanical adaptations after fatiguing exercise compared to more experienced runners [41]. For this reason, our results may not necessarily apply to novice runners and only apply to runners with a certain expertise in facing terrain irregularities. The variability of muscle activations can be even more affected in novice runners, underlying the greater need to understand the physiological effects induced by systematic surface-specific training and to define how variable-surface training helps injury prevention. Moreover, trails with uneven or prolonged steep banks may hinder forward locomotion, increase the risk of falls and cause lower extremity injuries in runners [42,43,44].

While neuromuscular demands did not increase in the experimental condition on average, the variability of muscle activations was significantly affected. Indeed, the coefficient of variation of muscle activation peak amplitudes increased by about 10%, and the width increased to about 8.3%. The repeated rolling of the treadmill forced runners to continuously adjust their motor strategies to the inclination of the surface. While those adjustments may have occurred within the boundaries of balance and comfort in our experienced sample of runners, they reflect a flexible modification of motor control strategies [20]. It is known that humans adjust their motor control strategies’ timing to deal with unsteady locomotion [20]; here, we suggest that this also occurs in the case of random rolling surfaces. However, future studies should assess the modular organization of muscle activation to better elucidate the demands imposed on motor control under rolling variations [19].

Even though cardiopulmonary, metabolic and neuromuscular variables were not affected by running conditions, they differed, as expected, between running speeds. This confirms the accuracy of the measurements made [30,32]. Similarly, the measured increase in sEMG activation was noted during higher running speeds, as the neuromuscular demand was previously shown to rise with increasing running intensity.

Since the specific physical demands of running can differ between surfaces, recreational and professional runners should resort to the use of non-traditional surfaces, emphasizing the specific surface-related motor tasks normally observed in natural running environments and, in particular, mountain trails.

## 5. Conclusions

The unpredictable roll variations (roll: ±7°; 10.4° s^−1^) used to reproduce a mountain pathway did not induce differences in cardiopulmonary or neuromuscular parameters in well-trained male athletes. However, the step-by-step variability of muscle activations was significantly affected.

Therefore, future studies should assess the modular organization of muscle activation and its cardio–metabolic–psychological effects on running on surfaces with unpredictable inclinations. Particular attention should be given to inexperienced or low-to-moderate fitness level athletes, as well as to faster speeds with respect to treadmill variations. It would allow the study of larger population effects and help understand how fitness level or individual characteristics impact these parameters and how external perturbation (given by faster treadmill variation) influences a runner’s performance.

## Figures and Tables

**Table 1:** Baseline characteristics of study participants.

Characteristics	*n* = 20
Age (years)	33 ± 8
Body mass (kg)	70.3 ± 6.4
Body height (m)	1.77 ± 0.06
BMI (kg/m^2^)	22.9 ± 1.9
GET (km·h^−1^)	13.0 ± 1.5
RCP (km·h^−1^)	15.0 ± 1.0
$\dot{V}$O_2_max (ml·kg^−1^·min^−1^)	63.8 ± 7.2
Training time (average/week, h)	10.5 ± 2.5
N° of training sessions/week	7 ± 1
Years competing in official races	5 ± 2

Data are expressed as mean ± standard deviation. *n*, number of subject; BMI, Body mass index; $\dot{V}$O_2_max, maximum oxygen uptake; GET, gas exchange threshold; RCP, respiratory compensation point.

**Table 2:** Metabolic measurements, gait and psychological parameters divided by running condition and speed.

			8 km·h^−1^	10 km·h^−1^	12 km·h^−1^
***Metabolic and cardiopulmonary measurements***					
$\dot{V}$ **O_2_**	*mlO_2_*·*kg^−1^*·*min^−1^*	FC	36.61 ± 2.83	43.85 ± 3.69 ^$$$^	51.33 ± 4.73 ^###, £££^
		URV	36.20 ± 2.67	43.16 ± 2.92 ^$$$^	51.57 ± 3.24 ^###, £££^
			p_bonf_ = 1, d = 0.045	p_bonf_ = 1, d = 0.116	p_bonf_ = 1, d = −0.180
**BLa^−^**	*mmol*·*L^−1^*	FC	1.35 ± 0.43	1.81 ± 0.78	3.49 ± 1.82
		URV	1.31 ± 0.45	1.80 ± 1.02	3.86 ± 2.25
			p_fried_ = 0.604		
**HR**	*bpm*	FC	126 ± 11	143 ± 13 ^$$$^	159 ± 14 ^###, £££^
		URV	127 ± 5	143 ± 18 ^$$$^	161 ± 17 ^###, £££^
			p_bonf_ = 1, d = −0.089	p_bonf_ = 1, d = −0.050	p_bonf_ = 1, d = −0.114
**Cr**	*J*·*kg^−1^*·*m^−1^*	FC	4.85 ± 0.48	4.79 ± 0.49	4.75 ± 0.47
		URV	4.82 ± 0.46	4.73 ± 0.38	4.82 ± 0.31
			p_bonf_ = 1, d = 0.071	p_bonf_ = 1, d = 0.146	p_bonf_ = 1, d = −0.155
***Gait parameters***					
**Ground time**	*ms*	FC	290.2 ± 20.6	258.2 ± 13.0 ^$$$^	234.4 ± 11.6 ^###, £££^
		URV	293.0 ± 17.5	258.8 ± 13.4 ^$$$^	235.0 ± 11.4 ^###, £££^
			p_bonf_ = 1, d = 0.011	p_bonf_ = 1, d = −0.014	p_bonf_ = 1, d = −0.019
**Cadence**	*spm*	FC	162.4 ± 9.9	165.8 ± 9.6 ^$$$^	170.4 ± 9.8 ^###, £££^
		URV	162.9 ± 10.0	168.0 ± 10.3 ^$$$^	173.2 ± 10.2 ^###, £££^
			p_bonf_ = 1, d = −0.017	p_bonf_ = 1, d = −0.088	p_bonf_ = 1, d = −0.065
***EMG parameters***					
**Peaks**	*mV*	FC	0.267 ± 0.038	0.247 ± 0.026	0.267 ± 0.04 ^#^
		URV	0.261 ± 0.066	0.161± 0.063	0.282 ± 0.034
			p_bonf_ = 1, d = −0.186	p_bonf_ = 1, d = −0.166	p_bonf_ = 1, d = −0.108
**CoV Pks**	*%*	FC	22 ± 4	19 ± 1	19 ± 1
		URV	24 ± 6	21 ± 5	22 ± 4
			p_bonf_ = 0.694, d = −0.226	p_bonf_ = 0.896, d = −0.213	p_bonf_ = 0.146, d = −0.297
**Width**	*ms*	FC	110 ± 8	108 ± 8 ^$$^	107 ± 8 ^###^
		URV	112 ± 8	109 ± 8 ^$$^	108 ± 7 ^###^
			p_bonf_ = 1, d = −0.171	p_bonf_ = 1, d = −0.141	p_bonf_ = 1, d = −0.092
**Cov Width**	*%*	FC	19 ± 2	18 ± 2	17 ± 2
		URV	20 ± 1	19 ± 2	19 ± 1
			p_bonf_ = 0.059, d = −0.256	p_bonf_ = 0.014 *, d = −0.301	p_bonf_ = 0.003 **, d = −0.342
***Psychological parameters***					
**RPE**		FC	9 ± 7	19 ± 13	35 ± 14
		URV	11 ± 9	22 ± 12	40 ± 16
			p_fried_ = 0.454		

Data are expressed as mean ± SD. FC, flat condition; URV, unpredictable roll variations. pbonf = *p*-value based on Bonferroni correction; pfried = *p*-value based on Friedman test. * = *p* < 0.05 FC vs. URV; ** = *p* < 0.01 FC vs. URV; ^#^ = *p* < 0.05 8 km·h^−1^ vs. 12 km·h^−1^; ^###^ = *p* < 0.001 8 km·h^−1^ vs. 12 km·h^−1^; ^$$^ = *p* < 0.01 8 km·h^−1^ vs. 10 km·h^−1^; ^$$$^ = *p* < 0.001 8 km·h^−1^ vs. 10 km·h^−1^; ^£££^ = *p* < 0.001 10 km·h^−1^ vs. 12 km·h^−1^.

## Data Availability

Data are available on reasonable request. All data relevant to the study are included in the article or uploaded as supplementary information (Supplementary Table S1).

## Supplementary Materials

The following are available online at https://www.mdpi.com/article/10.3390/ijerph20065189/s1, Table S1: Descriptive table with the results for each muscle and condition.
