# Supplementary material for: Flat versus Simulated Mountain Trail Running: A Multidisciplinary Comparison in Well-Trained Runners
Source: Int J Environ Res Public Health. 2023 Mar 15;20(6):5189. doi: 10.3390/ijerph20065189 (PMC10049634; doi:10.3390/ijerph20065189)
Supplement: Supplementary file 1 [file ijerph-20-05189-s001.zip › ijerph-2222905-supplementary.pdf]

Table S1. – Descriptive table with the results for each muscle and condition. Data are expressed as mean ± SD. FC, flat condition; URV, unpredictable roll variations; BF, bicep femoris; CoV, coefficient of variation; GM, gastrocnemius medialis; PL, peroneus longus; SOL, soleus; TA, tibialis anterior; VL, vastus lateralis; VM, vastus medialis. * = p<.05 FC vs URV; ** = p<.01 FC vs URV; *** = p<.001 FC vs URV; # = p<.05 8 $km{\cdot}h^{-1}$ vs. 12 $km{\cdot}h^{-1}$; ## = p<.01 8 $km{\cdot}h^{-1}$ vs. 12 $km{\cdot}h^{-1}$; ### = p<.001 8 $km{\cdot}h^{-1}$ vs. 12 $km{\cdot}h^{-1}$; $ = p<.05 8 $km{\cdot}h^{-1}$ vs. 10 $km{\cdot}h^{-1}$; $$ = p<.01 8 $km{\cdot}h^{-1}$ vs. 10 $km{\cdot}h^{-1}$; $$$ = p<.001 8 $km{\cdot}h^{-1}$ vs. 10 $km{\cdot}h^{-1}$; £ = p<.05 10 $km{\cdot}h^{-1}$ vs. 12 $km{\cdot}h^{-1}$; ££ = p<.01 10 $km{\cdot}h^{-1}$ vs. 12 $km{\cdot}h^{-1}$; £££ = p<.001 10 $km{\cdot}h^{-1}$ vs. 12 $km{\cdot}h^{-1}$

			**8 $km{\cdot}h^{-1}$**	**10 $km{\cdot}h^{-1}$**	**12 $km{\cdot}h^{-1}$**
**BF**	**Mean peaks (mV)**	**FC**	0.225±0.064	0.261±0.086	0.283±0.092
		**URV**	0.297±0.234	0.285±0.206	0.260±0.069
			p_{bonf} = 1, d = -0.503	p_{bonf} = 1, d = -0.168	p_{bonf} = 1, d = 0.162
	**Mean width (ms)**	**FC**	102±14	100±13	102±11
		**URV**	104±15	101±13$^{\$\$}$	104±12$^{£££}$
			p_{bonf} = 1, d = -0.141	p_{bonf} = <.001***, d = -1.279	p_{bonf} = 1, d = -0.175
**GM**	**Mean peaks (mV)**	**FC**	0.392±0.065	0.406±0.094	0.410±0.088
		**URV**	0.411±0.154	0.420±0.129	0.543±0.260$^{\#\#}$
			p_{bonf} = 1, d = -0.038	p_{bonf} = 1, d = -0.194	p_{bonf} = 0.042*, d = -0.906
	**Mean width (ms)**	**FC**	123±10	127±13	127±16
		**URV**	128±11	125±15	118±17$^{\#}$
			p_{bonf} = 1, d = -0.344	p_{bonf} = 1, d = 0.124	p_{bonf} = 0.102, d = 0.653
**PL**	**Mean peaks (mV)**	**FC**	0.217±0.078	0.230±0.084	0.246±0.079$^{\#\#\#}$
		**URV**	0.210±0.062	0.200±0.059	0.229±0.072$^{\#\#\#,£}$
			p_{bonf} = 1, d = 0.244	p_{bonf} = 1, d = 0.278	p_{bonf} = 1, d = 0.235
	**Mean width (ms)**	**FC**	115±14	113±15	110±15
		**URV**	119±12	119±13	117±15
			p_{bonf} = 1, d = -0.313	p_{bonf} = 0.278, d = -0.425	p_{bonf} = 0.203, d = -0.448
**SOL**	**Mean peaks (mV)**	**FC**	0.228±0.085	0.242±0.089	0.268±0.108$^{\#\#}$
		**URV**	0.270±0.146	0.268±0.133	0.290±0.144
			p_{bonf} = 1, d = -0.356	p_{bonf} = 1, d = -0.216	p_{bonf} = 1, d = -0.180
	**Mean width (ms)**	**FC**	120±9	119±12	116±12
		**URV**	118±11	116±11	116±11
			p_{bonf} = 1, d = 0.194	p_{bonf} = 1, d = 0.217	p_{bonf} = 1, d = -0.002
**ST**	**Mean peaks (mV)**	**FC**	0.221±0.078	0.235±0.051	0.298±0.088
		**URV**	0.305±0.269	0.312±0.206	0.291±0.125
			p_{bonf} = 0.980, d = -0.537	p_{bonf} = 1, d = -0.494	p_{bonf} = 1, d = 0.048
	**Mean width (ms)**	**FC**	103±11	103±12	104±12
		**URV**	107±12	104±14	104±14
			p_{bonf} = 0.467, d = -0.302	p_{bonf} = 1, d = -0.135	p_{bonf} = 1, d = -0.003
**TA**	**Mean peaks (mV)**	**FC**	0.166±0.083	0.176±0.091	0.179±0.057
		**URV**	0.159±0.075	0.175±0.081	0.176±0.054
			p_{bonf} = 1, d = 0.089	p_{bonf} = 1, d = 0.008	p_{bonf} = 1, d = 0.047

	**Mean width (ms)**	**FC**	102±17	96±15	99±16
		**URV**	105±14	104±18	103±16
			p_{bonf} = 1, d = -0.216	p_{bonf} = 0.419, d = -0.466	p_{bonf} = 1, d = -0.251
**VL**	**Mean peaks (mV)**	**FC**	0.205±0.044	0.232±0.050	0.249±0.041$^{\#\#}$
		**URV**	0.242±0.088	0.240±0.063	0.253±0.068
			p_{bonf} = 0.224, d = -0.618	p_{bonf} = 1, d = -0.129	p_{bonf} = 1, d = -0.065
	**Mean width (ms)**	**FC**	107±9	100±9$^{\$\$\$}$	96±10$^{\#\#\#}$
		**URV**	105±9	100±9$^{\$}$	100±9$^{\#\#}$
			p_{bonf} = 1, d = 0.195	p_{bonf} = 1, d = -0.034	p_{bonf} = 0.417, d = -0.353
**VM**	**Mean peaks (mV)**	**FC**	0.254±0.149	0.220±0.070	0.209±0.066
		**URV**	0.206±0.067	0.218±0.071	0.211±0.084
			p_{bonf} = 0.874, d = 0.539	p_{bonf} = 1, d = -0.096	p_{bonf} = 1, d = 0.102
	**Mean width (ms)**	**FC**	107±7	102±6$^{\$\$}$	99±8$^{\#\#\#}$
		**URV**	110±10	104±10	102±10
			p_{bonf} = 1, d = 0.919	p_{bonf} = 1, d = -0.219	p_{bonf} = 1, d = -0.289
